# Facile One-Step Fabrication of 1T-Phase-Rich Bimetallic CoFe Co-Doped MoS_2_ Nanoflower: Synergistic Engineering for Bi-Functional Water Splitting Electrocatalysis

**DOI:** 10.3390/molecules30112343

**Published:** 2025-05-27

**Authors:** Xinyue Li, Yahui Song, Yiming Huang, Jihui Zhang, Siyu Wu, Wentao Zhang, Jin Wang, Xian Zhang

**Affiliations:** School of Materials Science and Engineering, Qilu University of Technology, Shandong Academy of Sciences, Jinan 250353, China; lixinyue1103@163.com (X.L.); huangyiming1106@163.com (Y.H.); beckhamzjh@163.com (J.Z.); wusiyu18310202768@163.com (S.W.); 18866461235@163.com (W.Z.); wangjinsdu@qlu.edu.cn (J.W.)

**Keywords:** CoFe co-doped MoS_2_, synergistic effect, bifunctional, water splitting

## Abstract

MoS_2_ has emerged as a highly promising catalyst for the hydrogen evolution reaction (HER) owing to its exceptional catalytic properties. However, there is a pressing need to further enhance its reactivity and integrate oxygen evolution reaction (OER) capabilities to facilitate its industrial implementation. In this context, a dual-metal doping approach presents a straightforward and effective strategy to achieve superior catalytic performance. Systematic characterization and electrochemical evaluations reveal that the synergistic effects of Co and Fe doping significantly enhance both HER and OER activities, demonstrating remarkable potential for practical applications in energy conversion and storage systems. The unique flower-like architecture of the material endows it with a substantially enlarged surface area, which significantly increases the exposure of active sites and facilitates enhanced catalytic activity. Specifically, it achieves the low overpotentials of −127 and 292 mV at 10 mA cm^−2^ for HER and OER in alkaline media, respectively, and demonstrates excellent stability over a 10 h test. This research provides valuable insights into the development of advanced materials capable of efficiently performing both HER and OER processes, paving the way for potential applications in sustainable energy technologies.

## 1. Introduction

The energy scarcity and environmental deterioration caused by the overconsumption of conventional fossil fuels have forced people to search for alternative clean energy resources [1,2,3,4]. Among these, hydrogen energy has emerged as one of the most promising and ideal energy carriers, garnering significant attention [5,6]. Electrochemical water splitting offers a solution for sustainable hydrogen production through the synergistic action of the hydrogen evolution reaction (HER) and the oxygen evolution reaction (OER) [7,8,9]. However, due to the sluggish reaction kinetics, the development of efficient catalysts is essential to reduce reaction barriers and enhance efficiency [10,11]. Currently, noble metal-based catalysts (Pt/Ir) are widely recognized as the most advanced catalysts due to their exceptional electrocatalytic activity. However, the substantial cost and restricted reserves of these pose significant obstacles to their widespread implementation on a large scale [12,13,14,15]. Currently, most catalysts exhibit single functionality for either HER or OER, making the development of bifunctional catalysts an urgent priority [12,16,17,18]. Hence, the exploration and development of low-cost high-efficiency bifunctional catalysts for electrocatalytic water splitting hold great significance.

In recent years, a diverse array of non-precious metal catalysts have been extensively investigated, encompassing transition metal sulfides [9,19,20,21], phosphides [22,23,24], oxides/hydroxides [16,25,26], nitrides [27,28], selenides [29,30,31], among others. Notably, transition metal chalcogenides such as MoS_2_ [32,33,34], WS_2_ [19,35], and CoS_2_ [20,36] have garnered significant attention owing to their cost-effectiveness and exceptional catalytic performance. In particular, MoS_2_ has been the focus of much attention due to its special two-dimensional structure, ample active sites, and notably low H^+^ adsorption Gibbs free energy, comparable to that of Pt-based catalysts [37,38]. MoS_2_ exhibits three typical crystal structures: 2H, 1T, and 3R. Among them, the common 2H phase is a semiconducting phase, the 1T phase is a metallic phase, and the semiconducting 3R phase is relatively rare. The 2H phase, which exhibits thermodynamic stability, suffers from low electronic conductivity and a limited quantity of active sites, resulting in poor catalytic activity for HER [39]. On the contrary, the 1T phase has exposed catalytic active sites on both the edges and basal planes, along with enhanced electronic conductivity, making it more suitable as a catalyst for electrocatalysis [40,41,42]. Consequently, the development of 1T-MoS_2_ holds great potential for improving the efficiency of water electrolysis.

Despite the undeniable advantages of MoS_2_ in electrocatalytic HER, modifications are still necessary to lower the reaction barrier and enhance performance. Commonly used methods include elemental doping, defect engineering, constructing heterostructures, and so on. For example, Gurusamy et al. prepared a kind of co-doped MoS_2_ nanoparticle as the electrode [43]. Theoretical calculations show that the ΔG_H*_ of pure MoS_2_ is 2.46 eV, indicating that it cannot bind H* strongly. However, for the co-doped MoS_2_, the value of ΔG_H*_ was found to decrease to −0.25 eV. This strongly suggests that the doping enhances the electrocatalytic activity of MoS_2_ by altering its electronic structure. Significantly, its OER catalytic efficiency remains insufficient. The current research on MoS_2_ predominantly focuses on improving its HER performance, with relatively few studies addressing OER enhancement [14,44,45]. However, from an industrial perspective, developing a bifunctional catalyst for HER and OER in the same electrolyte is crucial for cost-effective productivity. Element doping, particularly bimetallic element doping, stands out as a simple yet effective strategy, capable of fine-tuning the electronic structure of materials and boosting the performance of active sites [18,46,47,48]. Additionally, the synergistic effect of the bimetallic element components significantly plays a crucial role in modifying the electronic structure of the material, increasing the number of reaction active sites, and the optimization of the adsorption and desorption mechanisms of reaction intermediates. For instance, Muhammad Ajmal et al. synthesized an iron-doped nickel hydroxy cyanate compound, in which the doped iron atoms served as the primary active sites for catalytic activity [49]. Yajuan Pan et al. synthesized a B and Fe co-doped CoP [50]. It was shown by theoretical calculations that the energy barrier for the rate-determining step at the Fe site that was doped was rather low, signifying that the active site was truly located on the Fe site. Furthermore, Co is also a commonly employed doping element. Numerous studies have shown that doping with Co can modify the electronic structure of materials, accelerate the kinetics of water decomposition, and Co can also function as an active site for OER reactions. For example, the co-doped CuO catalyst prepared by Sieon Jung et al. reduced the potential by 180 mV compared with undoped CuO [51]. Morphological regulation of electrocatalysts is also a significant approach. To maximize the exposure of active sites to the electrolyte, it is crucial to obtain a morphology characterized by a small size and a large specific surface area [52].

In this study, we utilized a straightforward one-step hydrothermal synthesis method to develop a CoFe co-doped MoS_2_ electrocatalyst with a distinctive flower-like morphology. The co-doping of Co and Fe, along with their synergistic effect, markedly enhances the HER and OER performance of the material. The unique flower-like structure provides a significantly increased specific surface area, thereby achieving the maximization of active site exposure. Importantly, the CoFe-MoS_2_ catalyst exhibits superior electrochemical performance compared with both Co-MoS_2_ and Fe-MoS_2_, underscoring the effectiveness of bimetallic doping. In an alkaline electrolyte, CoFe-MoS_2_ demonstrates exceptional catalytic performance. At a current density of 10 mA cm^−2^, it achieves overpotentials of −127 mV for HER and 292 mV for OER. Moreover, it maintains excellent performance during the 10 h stability test, with only a negligible decrease in activity.

## 2. Results and Discussion

### 2.1. Structure and Morphology Characterizations

**Figure 1:**
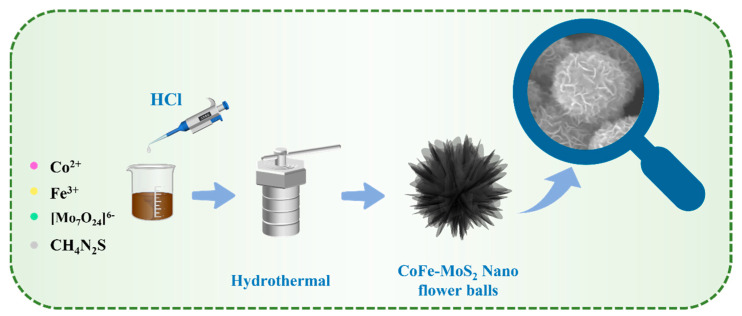
Schematic illustration of the synthesis procedure of CoFe-MoS_2_.

Figure 1 shows the preparation process of CoFe co-doped MoS_2_. CoFe-MoS_2_ was prepared by a simple one-step hydrothermal method, and in situ doping of Co and Fe was achieved by adding cobalt chloride and ferric chloride to the precursor solution. A series of MoS_2_, Co-MoS_2_, and Co_x_Fe_y_-MoS_2_ catalysts were prepared in order to investigate the effects of hydrochloric acid addition and heteroatom doping on MoS_2_. The detailed procedures are presented in the Section 3.

**Figure 2:**
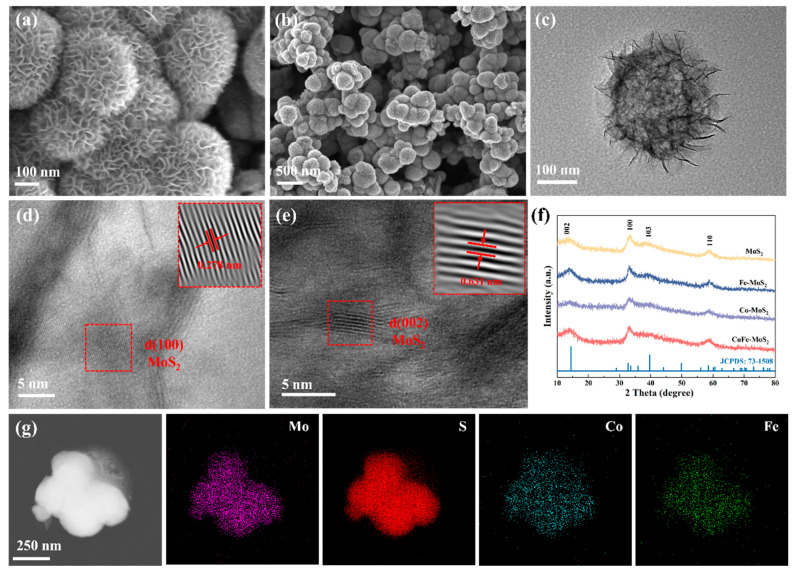
SEM images of (**a**,**b**) CoFe-MoS_2_. (**c**) TEM, (**d**,**e**) HRTEM images, (**f**) EDX element mapping images, and (**g**) XRD of CoFe-MoS_2_.

The morphology of the samples was thoroughly studied by scanning electron microscope (SEM). Figures S1 and S2 show the morphologies of MoS_2_ with different HCl additions. From the graph, it is clearly observed that, without the addition of HCl, the material appeared as irregular block-like shapes. When 1 mL of HCl was added, it exhibited a petal-like morphology. With the addition of 3 mL of HCl, the petal shapes were more complete and more regular. Notably, experimental optimization revealed that excessive HCl addition (5 mL) resulted in structural deformation characterized by the edge collapse of the petal-like formations. Therefore, we chose to add 3 mL of HCl as the basis for subsequent research, and added an appropriate amount of CoCl_2_·6H_2_O as a dopant on the basis of the original material to obtain a material with better performance. With the continuous increase in Co content, pyramid-shaped CoS_2_ appeared in the SEM image, as shown in Figure S3d, indicating that the doping of Co had reached its upper limit. Through a comparison of morphology and performance, we identified 15% as the optimal doping concentration. To further enhance catalytic performance, Fe was introduced as a synergistic dopant. By adjusting the ratio of Co and Fe, we obtained the final material. From Figure 2a,b and Figure S5, remarkably, the bimetallic doping process preserved the original petal-like architecture, yielding hierarchical flower-like microspheres with an average particle size of ~220 nm. These microspheres consisted of interconnected ultrathin nanosheets, creating a three-dimensional porous framework that maximized active site accessibility while maintaining structural integrity. TEM measurement was carried out to obtain more detailed information about the microstructure of the material. As shown in Figure 2c, the TEM image confirmed the hierarchical flower-like architecture, where individual petals were constructed from interconnected ultrathin nanosheets. The flower-like morphology endowed the material with a large surface area and exposed more active sites. The stacking of petals provided a multi-dimensional boundary, which is beneficial to the diffusion of electrolytes and accelerates mass transfer. In HRTEM, obvious lattice stripes of 0.278 nm and 0.631 nm were observed, corresponding to the (110) and (002) crystal planes of MoS_2_ (Figure 2d,e). Elemental mapping analysis demonstrated the homogeneous dispersion of Mo, S, Co, and Fe across the three-dimensional nanoflower architecture (Figure 2f), meaning that Co and Fe elements were successfully doped into MoS_2_. The crystalline structure of the fabricated samples was subjected to further characterization by means of X-ray diffraction (XRD). The XRD patterns of the synthesized samples had the same crystal phase structure. As shown in Figure 2g, all samples exhibited identical phase structures with characteristic diffraction peaks at 13.91°, 33.02°, 39.32°, and 58.73°, respectively, corresponding to the (002), (100), (103), and (110) planes of molybdenum disulfide (JCPDS No.73-1508) [53], confirming successful MoS_2_ synthesis. This shows that we successfully prepared MoS_2_. In addition, we found no obvious peak shift on the XRD pattern, which indicated that the crystal phase structure of the samples doped with Co, Fe element had not changed, and the successful doping of Co and Fe elements was achieved.

**Figure 3:**
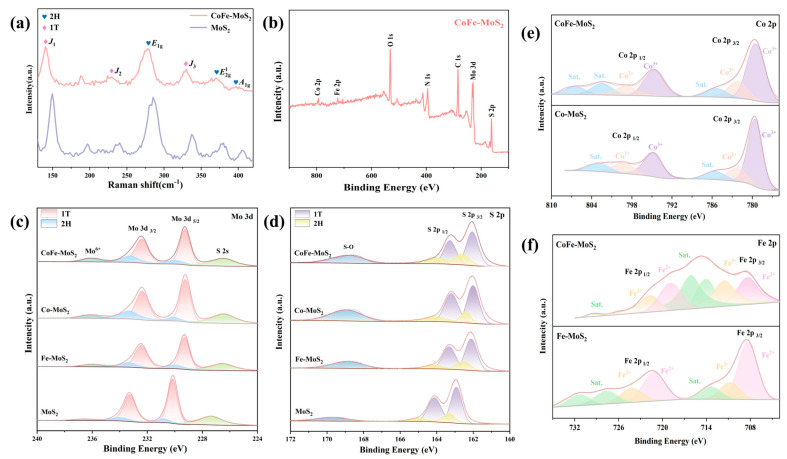
(**a**) Raman spectra, (**b**) overall XPS spectra of CoFe-MoS_2_, (**c**) Mo 3d, (**d**) S 2p, (**e**) Co 2p, and (**f**) Fe 2p.

Raman analysis was conducted to further determine the particular phase composition of the synthesized CoFe-MoS_2_ sample. Figure 3a shows the Raman spectra of MoS_2_ and CoFe-MoS_2_. Three characteristic peaks of 1T phase can be observed from the figure. The characteristic peaks of MoS_2_ were located at 149.6 (J_1_), 240.27 (J_2_), and 337.1 (J_3_) cm^−1^, and those of CoFe-MoS_2_ were located at 140.53 (J_1_), 229.45 (J_2_), and 329.99 (J_3_) cm^−1^, respectively [53,54]. At the same time, we also found characteristic peaks at 285.0 (E_1g_), 379.8 (E^1^_2g_), and 406.4 (A_1g_) cm^−1^ of MoS_2_ and 278.06 (E_1g_), 370.9 (E^1^_2g_), and 397.5 (A_1g_) cm^−1^ of CoFe-MoS_2_, respectively. This shows that 2H phase MoS_2_ also exists in the synthesized materials. The existence of 1T phase MoS_2_ improves the conductivity of the material, which is beneficial for the transmission of electrons and improves the catalytic efficiency of the material. The Raman spectra of Co-MoS_2_ and Fe-MoS_2_ also had similar characteristic peaks (Figures S6 and S7). Notably, the absence of significant peak shifts in the XRD patterns indicated that the crystal structure remained intact following Co and Fe doping. This structural preservation, combined with the uniform elemental distribution observed in EDS mapping, provides compelling evidence for the successful incorporation of Co and Fe dopants into the MoS_2_ lattice without inducing phase transformations or structural distortions [55,56,57].

X-ray photoelectron spectroscopy (XPS) was utilized to probe into the surface chemical states and elemental composition of the synthesized samples. The XPS measurement spectrum in Figure 3b revealed the existence of Fe, Mo, Co, S, O, N, and C elements, which proved the successful doping of Co and Fe elements. The existence of C and N elements was derived from the reactant TAA, and the existence of O element was attributed to the inevitable oxidation of materials exposed to air. Using the C 1s peak at 284.6 eV as a reference, all spectra were calibrated. As presented in Figure 3c, the Mo 3d spectrum could be accurately fitted into six peaks. In CoFe-MoS_2_, the peaks at 229.25 and 232.40 eV were attributed to Mo^4+^ 3d_5/2_ and Mo^4+^ 3d_3/2_ of 1T-MoS_2_, respectively. Meanwhile, the peaks at 229.98 and 233.24 eV referred to Mo^4+^ 3d_5/2_ and Mo^4+^ 3d_3/2_ of 2H-MoS_2_, proving the coexistence of 2H and 1T phases [44,54]. Additionally, the peak at 226.45 eV was assigned to S 2p, and the feature at 236.01 eV indicated the presence of Mo^6+^ species, likely resulting from surface oxidation [14]. Quantitative analysis of the Mo 3d spectra showed that the 1T phase accounted for 72.99% of the CoFe-MoS_2_ sample, while the 1T phase content in MoS_2_ was 66.63%, as determined by the integral area of the corresponding peaks [14,54]. This phenomenon suggests that the introduction of Co and Fe induces the generation of more 1T phases, which is conducive to the promotion of electron transport and the enhancement of the material’s intrinsic conductivity. The deconvoluted S 2p spectra provided further evidence of the existence of 1T and 2H phases of CoFe-MoS_2_ (Figure 3d). The peak detected at 168.85eV could be ascribed to SO_4_^2−^ species formed through surface oxidation [58]. Notably, compared with pure MoS_2_, the introduction of Co and Fe dopants induced a shift in the Mo 3d and S 2p XPS spectra of CoFe-MoS_2_, Co-MoS_2_, and Fe-MoS_2_ toward lower binding energies. This phenomenon indicates that the doping of Co and Fe leads to an increase in electron cloud density, which facilitates efficient charge transfer from the Co and Fe dopants to the Mo and S sites. Consequently, the observed interaction among Co, Fe, Mo, and S demonstrates that the doping of Co and Fe efficiently regulates the electronic structure of MoS_2_, and as a result, boosts the electrocatalytic activity of the material [47,59]. Figure 3e expounded the XPS spectra of Co 2p. In CoFe-MoS_2_, it was possible to see two spin–orbit peaks, which were the 2p_3/2_ and 2p_1/2_ peaks; in addition, three shake-up satellites (referred to as Sat.) could be identified. The peaks located at 794.65eV and 779.64 eV corresponded to the binding energies of the Co^3+^ 2p_1/2_ and 2p_3/2_ spin orbits, respectively. The peaks at 798.39 eV and 782.23 eV were assigned to Co^2+^, corresponding to Co^2+^ 2p_1/2_ and 2p_3/2_, respectively [54]. The incorporation of Co into the MoS_2_ lattice significantly modulated the local electronic structure, thereby activating the typically inert S atoms on the basal plane as efficient HER active sites. Figure 3f shows the Fe 2p XPS spectrum. In CoFe-MoS_2_, the peaks at 708.39 and 718.92 eV were attributed to the 2p_3/2_ and 2p_1/2_ states of Fe^2+^, respectively. Meanwhile, the peaks at 711.57 and 721.60 eV were associated with the 2p_3/2_ and 2p_1/2_ states of Fe^3+^ [55]. Quantitative analysis of the Fe 2p spectra revealed that the Fe^3+^ content in CoFe-MoS_2_ (fitted area: 875.85) was significantly higher than that in Fe-MoS_2_ (fitted area: 681.55), suggesting that the introduction of Co triggers the formation of more high-valence Fe species. These high-valence Fe species are particularly advantageous for OER catalysis, as they are expected to enhance the performance of materials in OER reaction [15,55]. It is noteworthy that both Co 2p and Fe 2p of CoFe-MoS_2_ exhibited a slight negative shift compared with Fe-MoS_2_ and Co-MoS_2_. This phenomenon suggests that Co and Fe can interact with each other, and the synergistic effect between the two can further regulate the redistribution of electrons in the material, which in turn enhances the catalytic activity.

### 2.2. Electrocatalytic Performance Investigation

To make a comprehensive appraisal of the electrocatalytic performance of the synthesized materials, we employed a conventional three-electrode electrochemical system. The catalytic activities of CoFe-MoS_2_, Fe-MoS_2_, Co-MoS_2_, and MoS_2_ were studied in a 1M KOH solution, with RDE supported by catalysts as the working electrode; the counter electrode was a graphite rod, and the reference electrode was an Ag/AgCl electrode, in order to reveal their performance of oxygen evolution reaction (OER) and hydrogen evolution reaction (HER).

**Figure 4:**
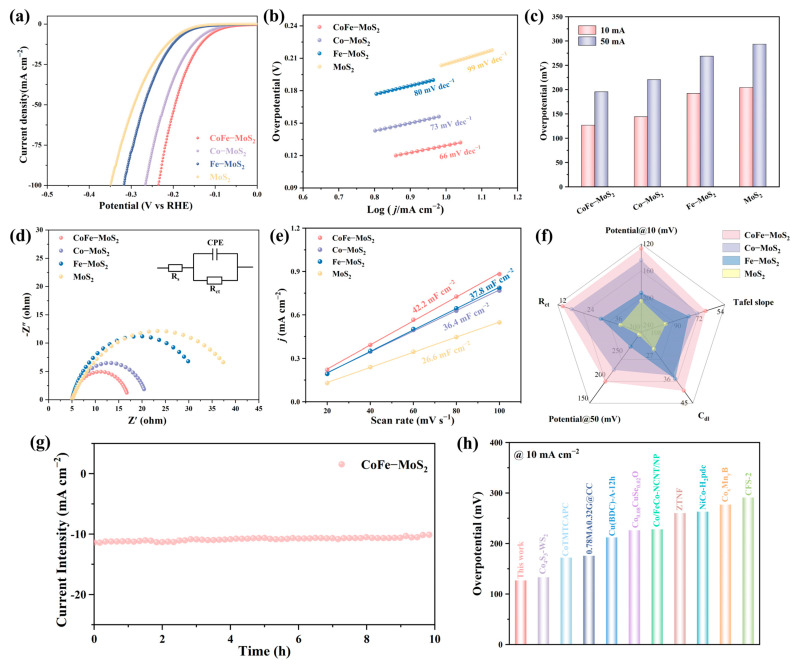
(**a**) HER polarization curve, (**b**) Tafel curve, (**c**) overpotential, (**d**) Nyquist plot, (**e**) electric double-layer capacitance of different materials. (**f**) Radar chart. (**g**) The potential versus time curve at a current density of 10 mA cm^−2^. (**h**) Comparison of HER overpotential with the recently reported electrocatalysts from Table S3.

The HER performance of the catalysts was systematically evaluated. As depicted in Figure 4a, CoFe-MoS_2_ demonstrated superior catalytic activity, requiring an overpotential of only 127 mV to achieve a current density of 10 mA cm^−2^, significantly outperforming Co-MoS_2_ (144 mV), Fe-MoS_2_ (192 mV), and pristine MoS_2_ (204 mV). This enhanced performance could be attributed to the synergistic effects of Co and Fe doping within the nanoflower architecture. Further optimization studies revealed that Co doping significantly improved HER activity, with 15% Co-MoS_2_ exhibiting the highest performance among various doping concentrations (Figure S8). Specifically, the η_10_ values for 5% Co-MoS_2_, 10% Co-MoS_2_, 25% Co-MoS_2_, and MoS_2_ were 178 mV, 167 mV, 156 mV, and 204 mV, respectively. That is, the optimal 15% Co-MoS_2_ sample attained an η_10_ value that was notably decreased. Specifically, this value was 29.4% lower than that of the MoS_2_ sample. In addition, the synergistic effect of Co and Fe needed to be in the right proportion to play the best role. The LSV curves of samples with different Co and Fe ratios are shown in Figure S9. The results show that the best performance and synergistic effect exerted maximum influence when Co:Fe was 1:1. The reaction kinetics of the HER were investigated further by means of the Tafel plots. As depicted in Figure 4b, it can be observed that CoFe-MoS_2_ exhibited the smallest Tafel slope (66 mV dec^−1^), which was lower than Co-MoS_2_ (73 mV dec^−1^), Fe-MoS_2_ (80 mV dec^−1^), and MoS_2_ (99 mV dec^−1^). The observation suggests that CoFe-MoS_2_ possesses greater advantages in terms of reaction kinetics [60]. Figure 4c shows that CoFe-MoS_2_ had the lowest overpotential at both 10 and 50 mA cm^−2^ current densities. As shown in Figure 4d, CoFe-MoS_2_ displayed much lower charge transfer resistance (R_ct_) than Co-MoS_2_, Fe-MoS_2_, and MoS_2_, which confirms that there is faster electron transfer and more excellent HER kinetics. The R_ct_ values are in Table S1. As shown in Figure 4e and Figure S10, we utilized cyclic voltammetry to measure the electrochemical double-layer capacitances (C_dl_) of diverse catalysts by cyclic voltammetry at different scanning rates in the non-Faraday region. Subsequently, we estimated the electrochemical surface areas (ECSA) of different catalysts based on the obtained C_dl_ values. Notably, the calculated C_dl_ values of CoFe-MoS_2_, Co-MoS_2_, Fe-MoS_2_, and MoS_2_ were 42.2, 37.8, 36.4, and 26.6 mF cm^−2^, respectively. Specific ECSA values are shown in Table S2. This suggests that Co and Fe doping increases the number of active sites and CoFe-MoS_2_ exposes more active sites for HER. A comprehensive performance comparison using radar charts (Figure 4f) incorporating multiple parameters (η_10_, η_50_, Tafel slope, C_dl_, R_ct_) confirmed the superior overall performance of CoFe-MoS_2_. Furthermore, Figure 4g illustrates the chronoamperometry curve of CoFe-MoS_2_ over 10 h. The results indicate that the current density of CoFe-MoS_2_ remained at 93.7% of the initial value after 10 h. As demonstrated in Figure 4h and Table S3, the HER overpotential achieved by our CoFe-MoS_2_ catalyst exhibited superior performance compared with numerous state-of-the-art electrocatalysts reported in the recent literature, showcasing its competitive advantage for practical hydrogen production applications.

**Figure 5:**
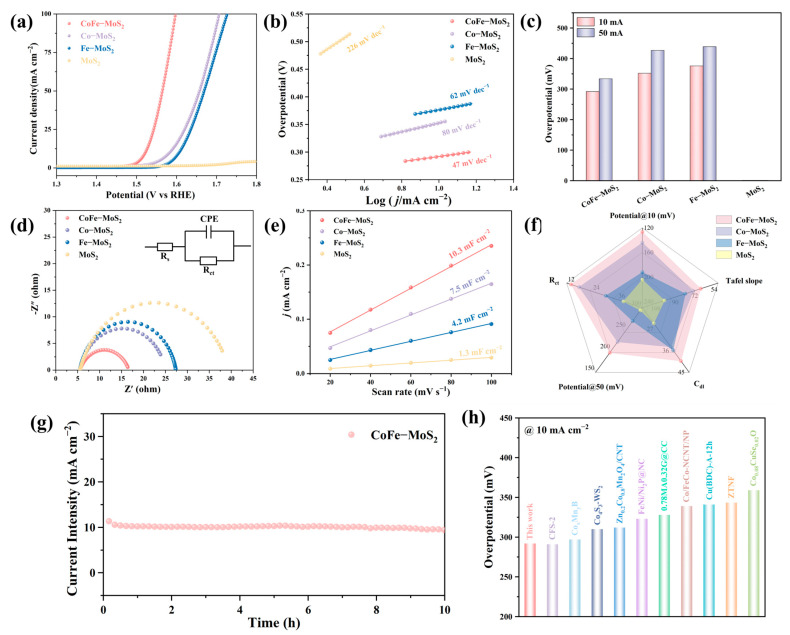
(**a**) OER polarization curve, (**b**) Tafel curve, (**c**) overpotential, (**d**) Nyquist plot, (**e**) electric double-layer capacitance of different materials. (**f**) Radar chart. (**g**) The potential versus time curve at a current density of 10 mA cm^−2^. (**h**) Comparison of OER overpotential with the recently reported electrocatalysts from Table S6.

Besides the HER, we also evaluated the OER performances of a range of catalysts while keeping the experimental conditions consistent. Systematic optimization revealed that the optimal OER performance was achieved at a Co doping level of 15% with a Co:Fe ratio of 1:1 (Figures S11 and S12), which was subsequently employed for further studies. Therefore, for the subsequent experiments, this Co doping level and the ratio of Co to Fe were selected. Figure 5a exhibits the linear sweep voltammetry (LSV) curves of CoFe-MoS_2_, Co-MoS_2_, Fe-MoS_2_, and MoS_2_, which indicate that CoFe-MoS_2_ displayed the best OER catalytic performance compared with others. Specifically, CoFe-MoS_2_ required an overpotential of only 292 mV to reach 10 mA cm^−2^, significantly lower than those of Co-MoS_2_ (352 mV), Fe-MoS_2_ (376 mV), and pristine MoS_2_. This remarkable enhancement highlights the critical importance of Co doping and the synergistic interplay between Co and Fe in boosting OER activity. Notably, undoped MoS_2_ showed essentially no OER activity, further confirming the necessity of dual-metal doping. To further assess the electron-transfer kinetics of electrocatalysts, the relevant Tafel plots are shown in Figure 5b. Notably, the Tafel slope value of CoFe-MoS_2_ was merely 47 mV dec^−1^. This value was significantly much lower than that of Co-MoS_2_ (80 mV dec^−1^), Fe-MoS_2_ (62 mV dec^−1^), and MoS_2_ (226 mV dec^−1^). This implies that CoFe-MoS_2_ has faster OER kinetics under the synergistic effect of Co and Fe. Figure 5c depicts the overpotentials of different catalysts when the current densities were 10 and 50 mA cm^−2^. In addition, the EIS results show the smallest charge transfer resistance (R_ct_) of CoFe-MoS_2_, much lower than that of Co-MoS_2_, Fe-MoS_2_, and MoS_2_, indicating optimal charge transfer characteristics during OER (Figure 5d). The R_ct_ values are in Table S4. Electrochemical surface areas are a vital criterion for assessing a catalyst. To compare the catalytic activity, the electrochemical double-layer capacitance (C_dl_) was subsequently measured (Figure 5e and Figure S13). The measured C_dl_ values were 10.3, 7.5, 4.2, and 1.3 mF cm^−2^ for CoFe-MoS_2_, Co-MoS_2_, Fe-MoS_2_, and MoS_2_, respectively, implying that CoFe-MoS_2_ possesses the most abundant active sites. The corresponding ECSA values are given in Table S5. As shown in the radar plot of Figure 5f, CoFe-MoS_2_ exhibited the most superior OER activity when in an alkaline solution environment. Furthermore, long-term stability tests (Figure 5g) demonstrated outstanding durability, with CoFe-MoS_2_ retaining 91.81% of its initial current density after 10 h of continuous operation. As evidenced in Figure 5h, the OER overpotential demonstrated by our catalyst exhibited superior performance compared with numerous state-of-the-art electrocatalysts recently reported in the literature, highlighting its competitive edge in practical water splitting systems. The corresponding specific data are given in Table S6.

## 3. Materials and Methods

### 3.1. Materials and Reagents

All the chemicals—ammonium molybdate tetrahydrate ((NH_4_)_6_Mo_7_O_24_, Aladdin, Shanghai, China), thioacetamide (TAA, C_2_H_5_NS, Maklin, Shanghai, China), cobalt chlo-ride hexahydrate (CoCl_2_·6H_2_O, Aladdin, Shanghai, China), anhydrous ferric chloride (FeCl_3_, Aladdin, Shanghai, China), hydrochloric acid (HCl, 37%, Fu Yu, Tianjin, China), anhydrous ethanol (analytical grade, Sinopharm, Shanghai, China), and potassium hy-droxide (KOH, Maklin, Shanghai, China)—were reagent grade and used as received without further purification. Ultrapure water was used throughout the experiments.

### 3.2. Synthesis of CoFe-MoS_2_

CoFe-MoS_2_ was synthesized via a hydrothermal treatment. Specifically, (NH_4_)_6_Mo_7_O_24_ (0.81 mmol), CoCl_2_·6H_2_O (0.42 mmol), and FeCl_3_ (0.42 mmol) were dissolved in ultrapure water and stirred for 15 min. Subsequently, TAA (33.3 mmol) and 3 mL HCl were added, followed by an additional 15 min of stirring. Subsequently, the obtained mixture was conveyed into a 50 mL Teflon-lined stainless steel autoclave. The autoclave was then held at a temperature of 250 °C for a duration of 24 h. Upon cooling to an ambient temperature, the black precipitate was collected by centrifugation and washed with ultrapure water and ethanol, and dried overnight at 60 °C. The final product was designated as CoFe-MoS_2_.

Co-MoS_2_, Fe-MoS_2_, and MoS_2_ were synthesized by the same method, but CoCl_2_·6H_2_O and FeCl_3_ were removed accordingly.

### 3.3. Electrochemical Characterization

Electrochemical measurements were conducted using a three-electrode configuration employing the CHI 660e electrochemical workstation at an ambient temperature. A 1 M KOH solution with a pH of 14 was employed as the electrolyte. A graphite rod electrode served as the counter electrode, an Ag/AgCl electrode was used as the reference electrode, and the working electrode was a glassy carbon electrode with a diameter of 5 mm that was coated with catalyst inks.

Preparation of working electrode: A certain amount of electrocatalyst (6 mg) and XC-72 carbon powder (1 mg) was dispersed in a mixed solution containing isopropanol (480 μL) and Nafion (25 μL, 5 wt%). Then, the obtained mixture was subjected to ultrasonic treatment for 0.5 h to obtain a uniform ink. Then, 10 μL of the solution was dripped onto the glass carbon electrode (0.196 cm^2^), and the required working electrode (catalyst loading of 0.6 mg cm^−2^) was obtained after natural drying.

The measured potential (E_Ag/AgCl_) was converted into reversible hydrogen potential (RHE) based on the following formula: E_RHE_ = 0.198V + 0.0592 × pH + E_Ag/AgCl_. HER and OER activities were evaluated on a rotating disk electrode with a rotating speed of 1600 rpm. Firstly, the glassy carbon electrode for HER and OER was measured 20 times by cyclic voltammetry at a scanning rate of 50 mV s^−1^, and the steady-state current was obtained. Linear sweep voltammetry (LSV) was measured at a scanning rate of 5 mV s^−1^, and 90% iR correction was performed to compensate. LSV was recorded in the range 0.2 to −0.5 V vs. RHE for HER and 1.2 to 1.8 V for OER. OER overpotential (η) was calculated by the following formula: η = ERHE − 1.23. Tafel slope was obtained from LSV data by the following formula: η = a × log |j| + b, where “a” stands for Tafel slope, “b” stands for intercept, and “j” stands for current density. Electrochemical active surface area (ECSA) was measured by studying the electrochemical double-layer capacitance (C_dl_) in the Faraday region. Cyclic voltammetry (CV) was carried out at different scan rates (20, 40, 60, 80, 100 mV s^−1^) in the non-Faradaic potential range to estimate C_dl_. By calculating the half slope (vs. RHE) of the current density difference at different scanning rates, the C_dl_ value was obtained. The equation for calculating the electrochemically active surface area (ECSA) is expressed as ECSA = C_dl_/C_s_. Here, C_s_ represents the ideal specific capacitance of the sample. In an alkaline solution, it is selected as an average value of 40 μF. Electrochemical impedance spectroscopy (EIS) was performed using a potential disturbance with an amplitude of 5 mV in the frequency range of 100,000 Hz to 0.1 Hz. To evaluate stability, chronoamperometric measurement curves were carried out at a constant current density.

### 3.4. Characterization

Scanning electron microscopy (SEM, Gemini, 500-70-89, Carl Zeiss, Oberkochen, Germany) and transmission electron microscopy (TEM, JEOL JEM 2100F, Tokyo, Japan) were employed to characterize the morphology and microstructure of the samples. The crystal diffraction patterns of samples were recorded by X-ray diffractometer (XRD, Smartlab SE, Rigaku, Tokyo, Japan) equipped with a Cu Kα radiation source (λ = 1.5418 Å). The surface composition and valence state of the samples were characterized by X-ray photoelectron spectroscopy (XPS, Thermo Scientific Escalab 250Xi, Santa Clara, CA, USA). Raman spectra (Raman) maps were obtained by an LabRAM HR Evolution Raman spectrometer (HORIBA, Villeneuve d’Ascq, France).

## 4. Conclusions

In summary, we successfully prepared Co and Fe co-doped flower-like MoS_2_ nanostructures through a facile one-step hydrothermal method. The hierarchical flower-like architecture endows the material with a substantially enlarged specific surface area, maximizing the exposure of catalytically active sites. Notably, the material exhibits a high 1T-phase content (72.99%), which significantly enhances its electrical conductivity and facilitates rapid electron transfer during electrochemical processes. Doping the material with Co and Fe, and the resulting synergistic effect, have greatly enhanced its performance in both the HER and the OER. The optimized catalyst achieves remarkably low overpotentials of −127 and 292 mV at 10 mA cm^−2^ for HER and OER in alkaline media, coupled with robust stability over 10 h of continuous operation. This study provides a new idea for the preparation of HER and OER bifunctional catalysts with bimetallic synergistic effects and provides insights into exploring efficient catalysts for energy conversion technologies.

## Data Availability

The data supporting this article have been included as part of the Supplementary Information.

## Supplementary Materials

The following supporting information can be downloaded at: https://www.mdpi.com/article/10.3390/molecules30112343/s1, Figure S1. (a,b) SEM images of the MoS_2_-3mL HCl; Figure S2. SEM image of the (a,d) MoS_2_-0 mL HCl, (b,e) MoS_2_-1 mL HCl, and (c,f) MoS_2_-5 mL HCl; Figure S3. SEM image of the Co-MoS_2_ with different Co ratios (a) 5%, (b) 10%, (c) 15%, and (d) 25%; Figure S4. SEM image of the CoFe-MoS_2_ with different CoFe ratios (a) Co:Fe = 1:4, (b) Co:Fe = 4:1; Figure S5. SEM image of the 15%Fe-MoS_2_; Figure S6. Raman of Co-MoS_2_; Figure S7. Raman of Co-MoS_2_; Figure S8. HER polarization curves for the MoS_2_ with different Co doping amounts; Figure S9. HER polarization curves for the MoS_2_ with different Co and Fe ratios; Figure S10. C_dl_ measurements with different scanning rates of 20, 40, 60, 80, and 100 mV s^–1^ for (a) CoFe-MoS_2_, (b) Co-MoS_2_, (c) CoFe-MoS_2_, and (d) MoS_2_; Figure S11. HER polarization curves for the MoS_2_ with different Co doping amounts; Figure S12. OER polarization curves for the MoS_2_ with different Co and Fe ratios; Figure S13. C_dl_ measurements with different scanning rates of 20, 40, 60, 80, and 100 mV s^–1^ for (a) CoFe-MoS_2_, (b) Co-MoS_2_, (c) CoFe-MoS_2_, and (d) MoS_2_; Table S1. The R_ct_ value of different catalysts; Table S2. The ECSA value of different catalysts; Table S3. Comparison of HER performance of CoFe-MoS_2_ with other electrocatalysts reported previously in 1 M KOH; Table S4. The R_ct_ value of different catalysts; Table S5. The ECSA value of different catalysts; Table S6. Comparison of OER performance of CoFe-MoS_2_ with other electrocatalysts reported previously in 1 M KOH. References [61,62,63,64,65,66,67,68,69,70,71,72] are cited in Supplementary Materials

## Abbreviations

The following abbreviations are used in this manuscript:

TAA	Thioacetamide
