# Supplementary material for: Facile One-Step Fabrication of 1T-Phase-Rich Bimetallic CoFe Co-Doped MoS2 Nanoflower: Synergistic Engineering for Bi-Functional Water Splitting Electrocatalysis
Source: Molecules. 2025 May 27;30(11):2343. doi: 10.3390/molecules30112343 (PMC12156378; doi:10.3390/molecules30112343)
Supplement: Supplementary file 1 [file molecules-30-02343-s001.zip › molecules-3606370-supplementary.pdf]

Supporting Information

Facile One-Step Fabrication of 1T-Phase-Rich Bimetallic CoFe Co-Doped MoS_2 Nanoflower: Synergistic Engineering for Bifunctional Water Splitting Electrocatalysis

Xinyue Li, Yahui Song,* Yiming Huang, Jihui Zhang, Siyu Wu, Wentao Zhang, Jin Wang* and Xian Zhang*

School of Materials Science and Engineering, Qilu University of Technology (Shandong Academy of Sciences), Jinan, 250353, P. R. China.

E-mail: songyahui@qlu.edu.cn

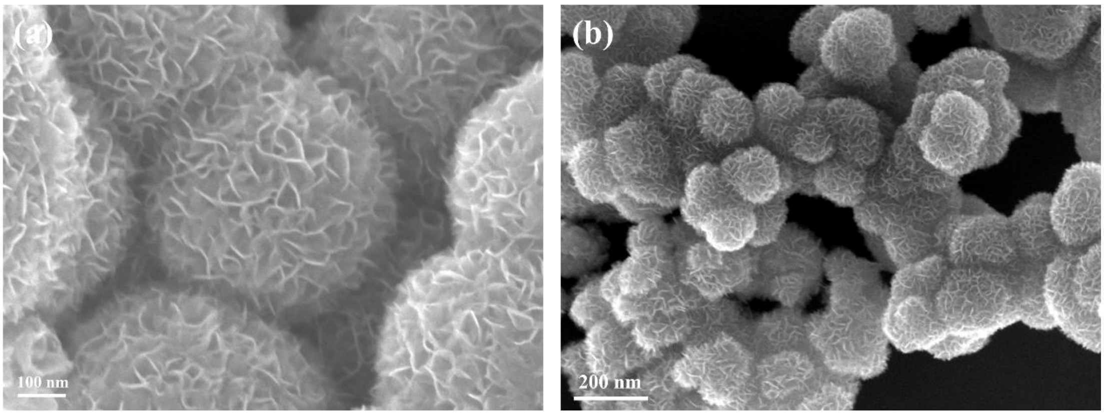

Figure S1. (a), (b) SEM images of the MoS_2-3mL HCl.

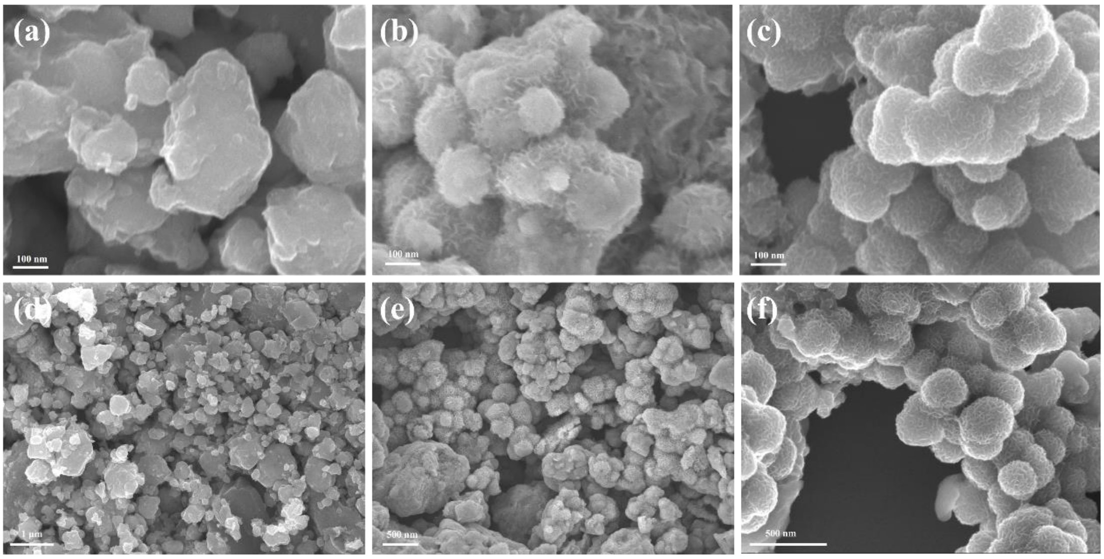

Figure S2. SEM image of the (a)(d) MoS_2-0 mL HCl, (b)(e) MoS_2-1 mL HCl, and (c)(f) MoS_2-5 mL HCl.

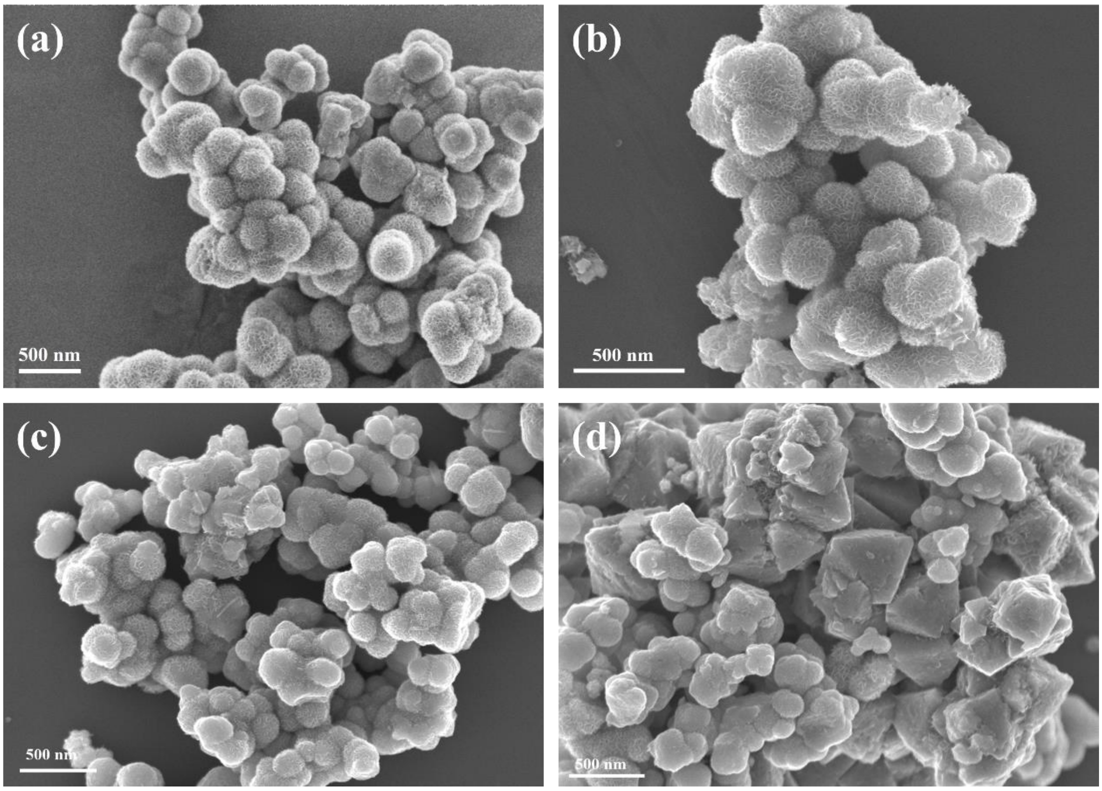

Figure S3. SEM image of the Co-MoS_2 with different Co ratios (a)5%, (b)10%, (c)15%, and (d) 25%.

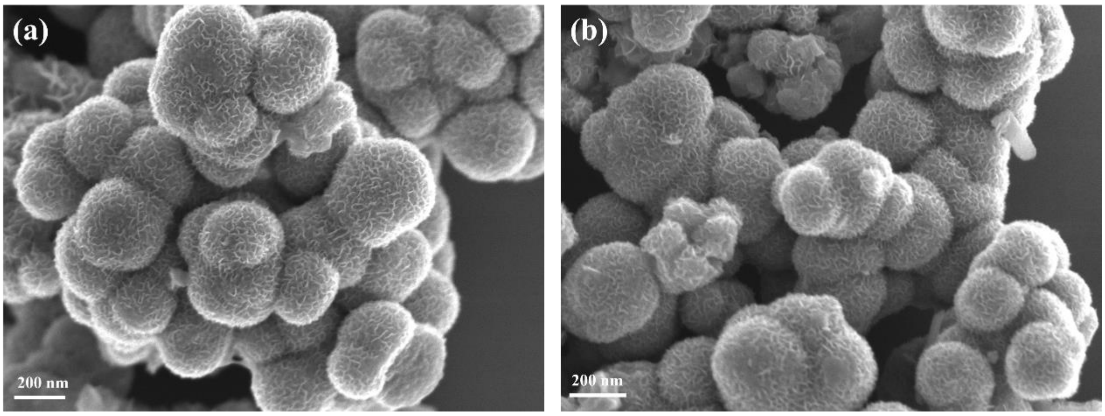

Figure S4. SEM image of the CoFe-MoS_2 with different CoFe ratios (a)Co:Fe=1:4, (b) Co:Fe=4:1.

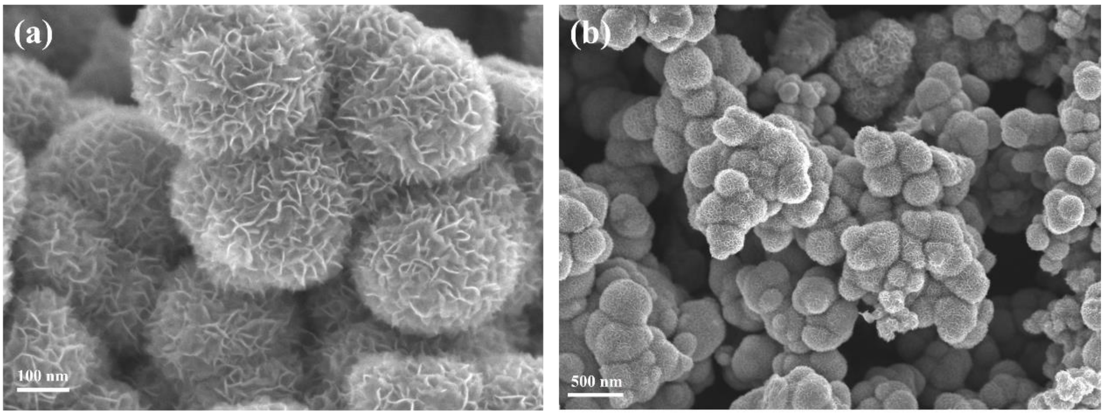

Figure S5. SEM image of the 15%Fe-MoS_2.

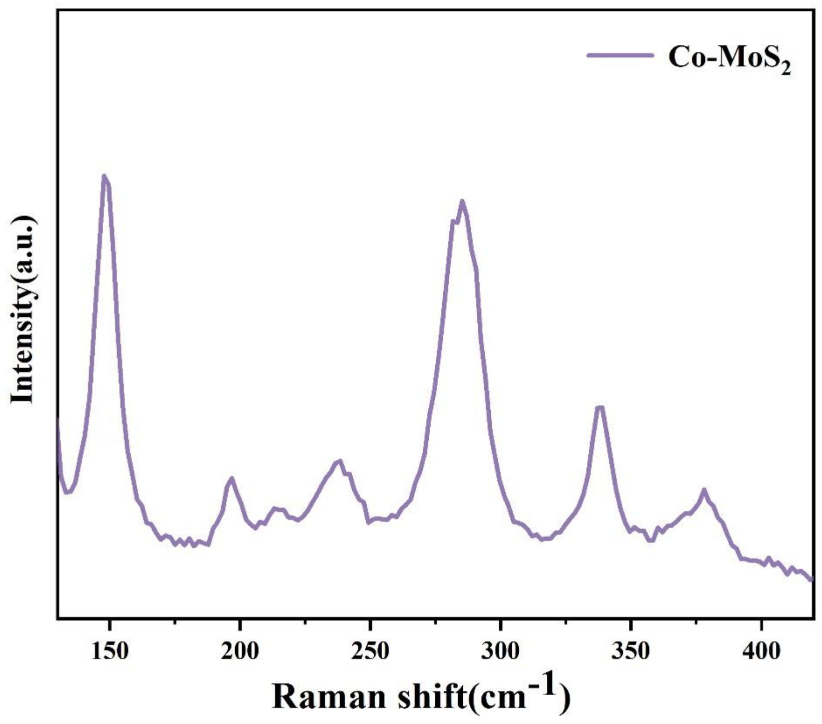

Figure S6. Raman of Co-MoS_2.

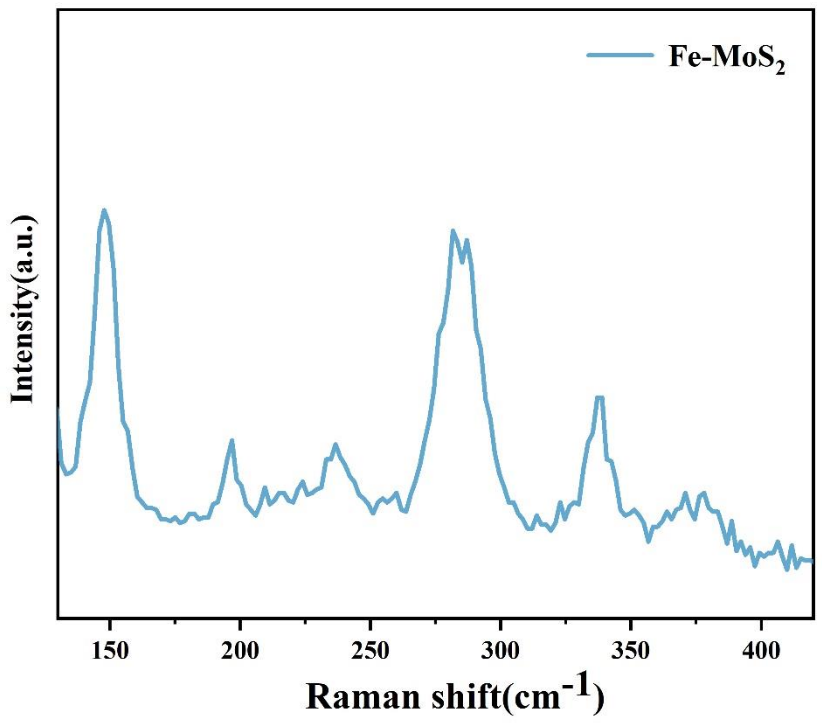

Figure S7. Raman of Co-MoS_2.

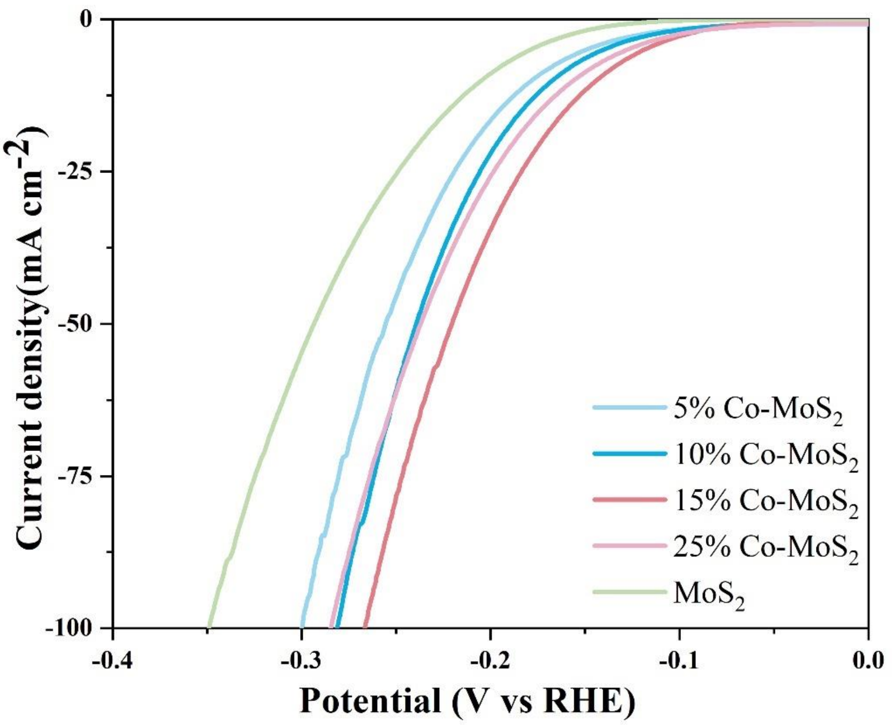

Figure S8. HER polarization curves for the MoS_2 with different Co doping amounts.

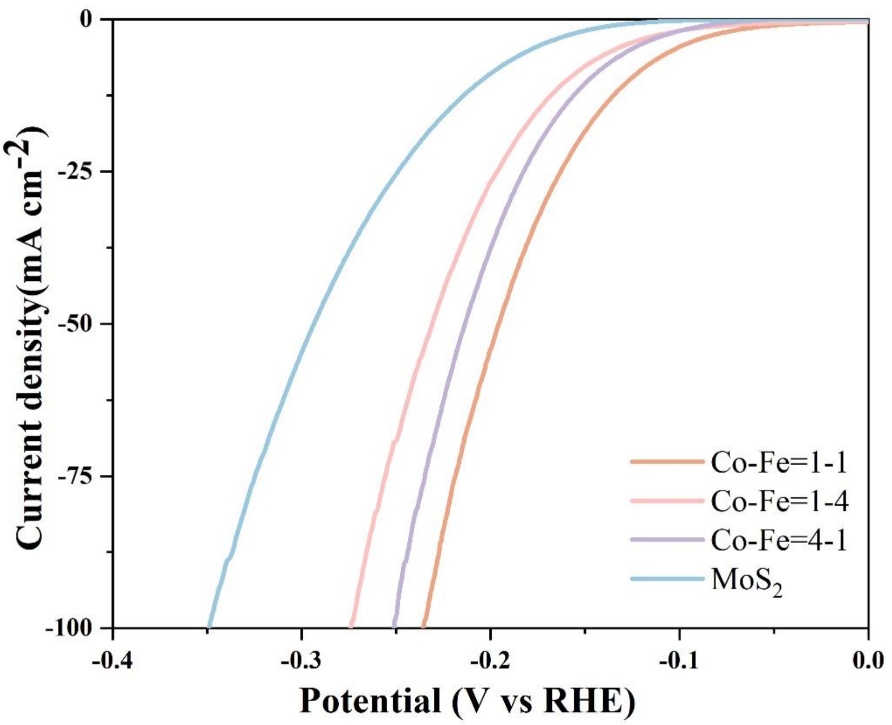

Figure S9. HER polarization curves for the MoS_2 with different Co and Fe ratios.

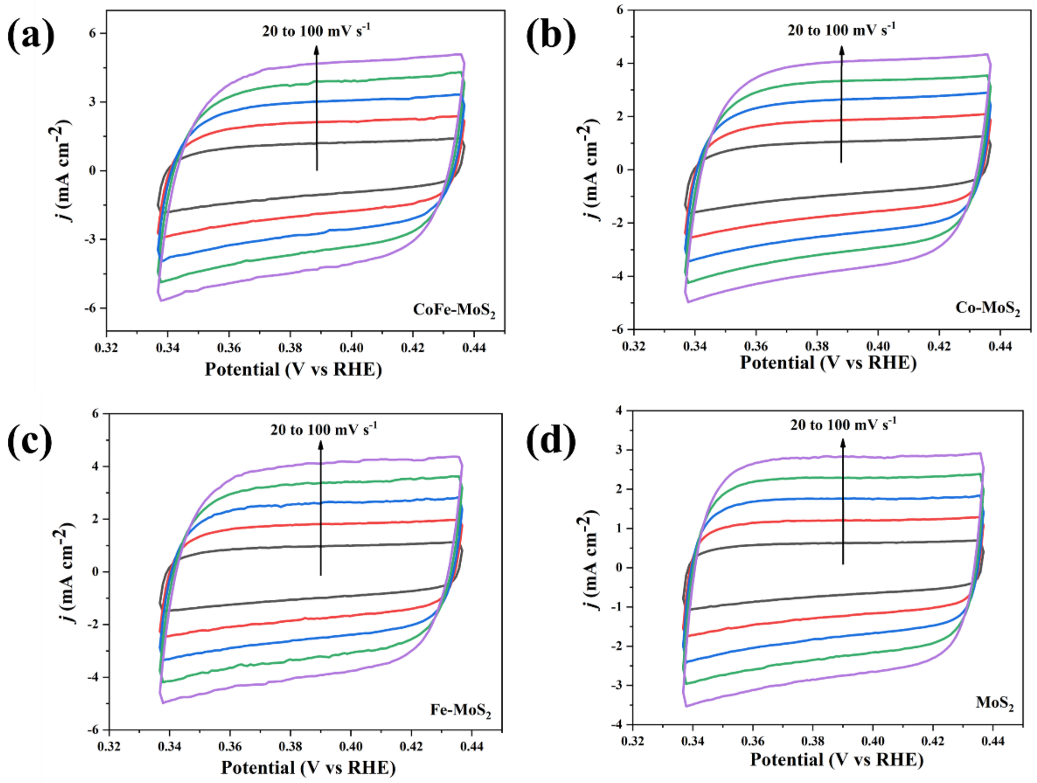

Figure S10. C_{dl} measurements with different scanning rates of 20, 40, 60, 80, and 100 mV s^{-1} for (a) CoFe-MoS_2, (b) Co-MoS_2, (c) CoFe-MoS_2, and (d) MoS_2.

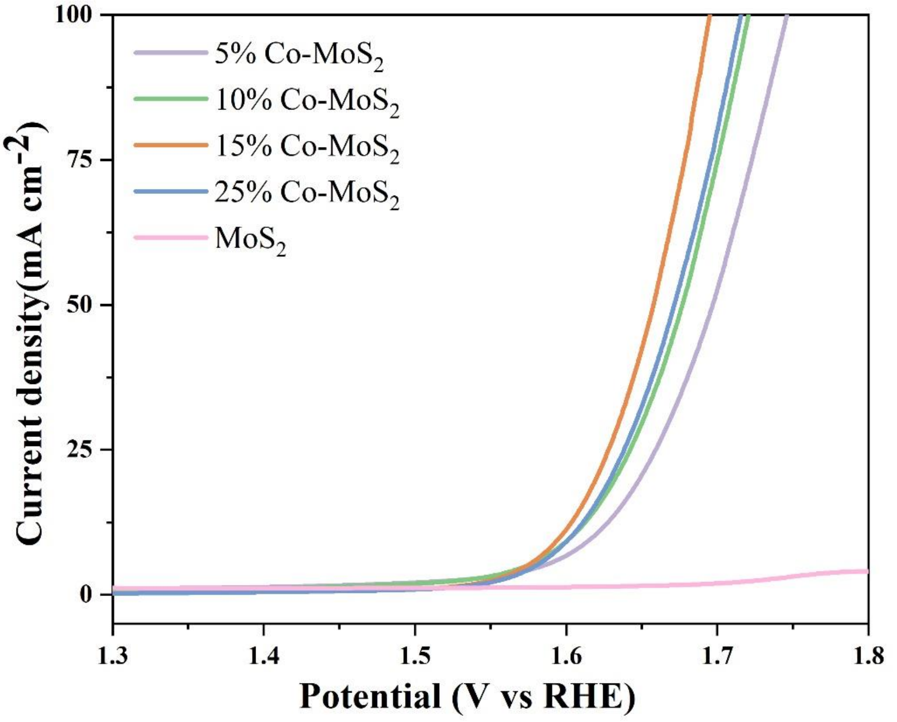

Figure S11. HER polarization curves for the MoS_2 with different Co doping amounts.

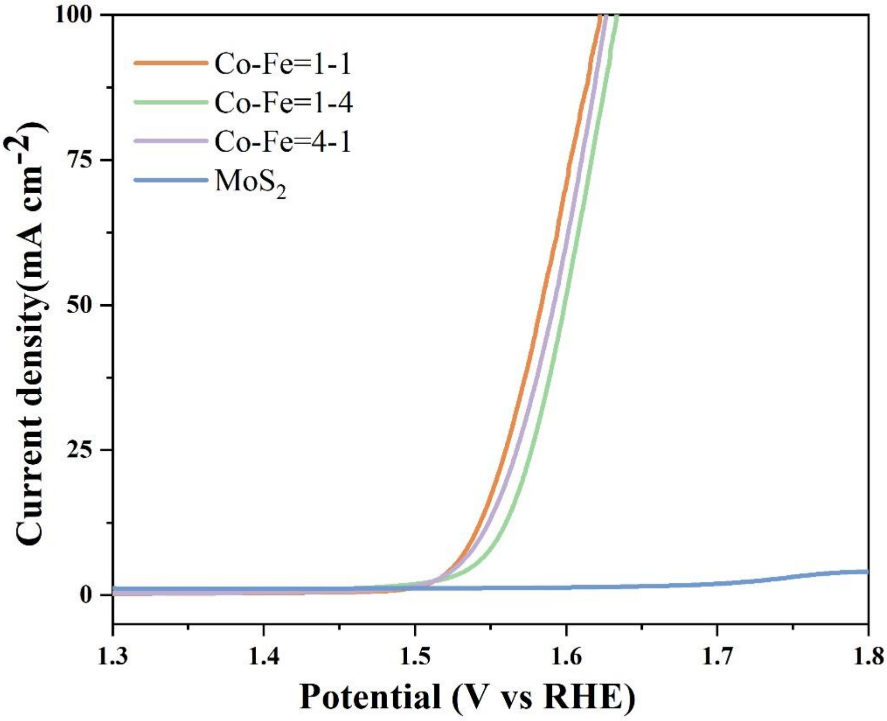

Figure S12. OER polarization curves for the MoS_2 with different Co and Fe ratios.

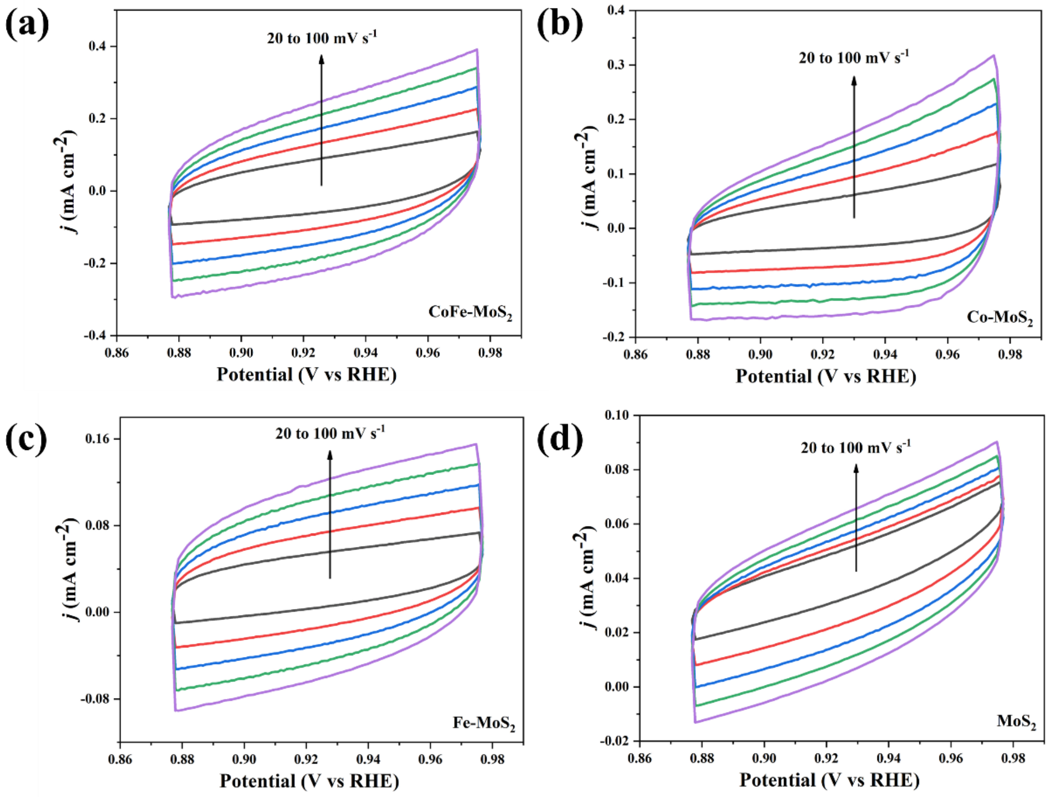

Figure S13. C_{dl} measurements with different scanning rates of 20, 40, 60, 80, and 100 mV s^{-1} for (a) CoFe-MoS_2, (b) Co-MoS_2, (c) CoFe-MoS_2, and (d) MoS_2.

Table S1. The R_{ct} value of different catalysts.

Sample	R_s/Ω	R_{ct}/Ω
CoFe-MoS_2	5.02	12.1
Co-MoS_2	5.089	15.97
Fe-MoS_2	5.054	28.62
MoS_2	5.064	37

R_s represents the solution resistance.

Table S2. The ECSA value of different catalysts.

Sample	ECSA
CoFe-MoS_2	1056 cm^2_{ECSA}
Co-MoS_2	943.75 cm^2_{ECSA}
Fe-MoS_2	910.5 cm^2_{ECSA}
MoS_2	664.5 cm^2_{ECSA}

Table S3. Comparison of HER performance of CoFe-MoS_2 with other electrocatalysts reported previously in 1 M KOH.

Sample	Overpotential (mV)	Tafel slope	Reference
CoFe-MoS_2	126.7	66.04	This work
Co_4S_3-WS_2	133	114	[61]
CoTMTCAPc	172	147	[62]
0.78MA0.32G@CC	175.6	45.5	[63]
Cu(BDC)-A-12h	212	115	[64]
$Co_{0.08}CuSe_{0.02}O$	226	148	[65]
Co/FeCo-NCNT/NP	228	99	[66]
ZTNF	260.3	123.5	[67]
NiCo-H_2pdc	263	73	[68]
Co_xMn_yB	277	71	[69]
CFS-2	291	164	[70]

Table S4. The R_{ct} value of different catalysts.

Sample	R_s/Ω	R_{ct}/Ω
CoFe-MoS_2	5.514	11.17
Co-MoS_2	5.523	19.54
Fe-MoS_2	5.54	21.99
MoS_2	5.536	33.95

R_s represents the solution resistance.

Table S5. The ECSA value of different catalysts.

Sample	ECSA
CoFe-MoS_2	256.25 cm^2_{ECSA}
Co-MoS_2	187.5 cm^2_{ECSA}
Fe-MoS_2	105.25 cm^2_{ECSA}
MoS_2	32.75 cm^2_{ECSA}

Table S6. Comparison of OER performance of CoFe-MoS_2 with other electrocatalysts reported previously in 1 M KOH.

Sample	Overpotential (mV)	Tafel slope	Reference
CoFe-MoS_2	292	47	This work
CFS-2	291	65	[70]
Co_xMn_yB	297	62	[69]
Co_4S_3-WS_2	310	55	[61]
$Zn_{0.2}Co_{0.8}Mn_2O_4$/CNT	312	58.4	[71]
FeNi/Ni_2P@NC	323	60.3	[72]
0.78MA0.32G@CC	328	77.9	[63]
Co/FeCo-NCNT/NP	339	86	[66]
Cu(BDC)-A-12h	341	236	[64]
ZTNF	343.4	41.7	[67]
$Co_{0.08}CuSe_{0.02}O$	359	97	[65]

Reference

61. Wang, J.; Ling, Q.; Yao, Y.; Zhu, D.; Shu, S.; Zhou, Z.; Wu, X.; Wu, P. Willow Catkin-like Co_4S_3-WS_2 Nanostructured Electrocatalyst for Efficient Overall Alkaline Water Splitting. *ACS Appl. Nano Mater.* **2024**, *7*, 24408–24416, doi:10.1021/acsanm.4c04004.

62. Mounesh; Thippeswamy, B.A.; Shiralkar, P.; Balakrishna, R.G.; Nagaraja, B.M.; Pramoda, K. Non-Precious Tetra-(4-Methylthiazole)-Carboxamide Cobalt(II) Phthalocyanine Supported on Functionalized Carbon Nanotubes as an Efficient Electrocatalyst for a Hydrogen Evolution Reaction. *ACS Appl. Energy Mater.* **2024**, acsaem.4c01292, doi:10.1021/acsaem.4c01292.

63. Cheng, T.; Chen, C.; Wen, M.; Pan, F.; Zhang, X.; Ma, H.; Hou, B.; Xin, X. Low-Cost Composite Electrodes by Active Fe_3O_4 (111) of Fly Ash Magnetic-Sphere for Efficient Electrochemical Overall Water Splitting. *Int. J. Environ. Sci. Technol.* **2024**, doi:10.1007/s13762-024-05744-z.

64. Shooshtari Gugtapeh, H.; Abbasi, M.; Hasanzadeh Moghadam, M.; Rezaei, M. Solvent-Exchange-Assisted Activation of Cu-1,4-Benzene Dicarboxylate Metal-Organic Framework for Use as a Bifunctional Water Splitting Electrocatalyst. *Electrochimica Acta* **2024**, *508*, 145224, doi:10.1016/j.electacta.2024.145224.

65. Rashid, U.; Zhu, Y.; Cao, C. Microwave Assisted Synthesis of Cobalt-Doped Copper Selenite Nanorice as Bifunctional Electrocatalyst for Overall Water Splitting. *Journal of Electroanalytical Chemistry* **2024**, *962*, 118267, doi:10.1016/j.jelechem.2024.118267.

66. Zhang, Y.; Liu, X.; Wan, Z.; Wang, Z.; Gao, F.; Xuan, C. Nitrogen-Doped Carbon Nanotubes/Nanoparticles Confined Co/FeCo Composites with Metal-Nitrogen Sites for Efficient Multifunctional Electrocatalysis. *Journal of Environmental Chemical Engineering* **2024**, *12*, 114326, doi:10.1016/j.jece.2024.114326.

67. Huang, C.; Zhan, G.; Xiao, Z.; Lin, S. Synergistic Dual-Functional Full Deionization and Electrocatalysis of Water by ZnO/$Ti_3C_2T_x$ Heterojunction Supported with Novel Template. *Next Materials* **2024**, *5*, 100267, doi:10.1016/j.nxmate.2024.100267.

68. Zahid, R.; Abdul Karim, M.R.; Khan, F.S.; Zeb, G.; Marwat, M.A.; Khan, M.Z.; Gohar, O.; Haq, E.U. Catalytically Active Bimetallic Nickel–Cobalt MOF Linked Via Pyridine 2, 6-Dicarboxylate for Electrochemical Water Splitting Applications. *Arab J Sci Eng* **2024**, doi:10.1007/s13369-024-09613-2.

69. Li, S.; Wang, Z.; Yang, Y.; Pan, S.; Pan, W.; Tang, M.; Liu, K. Electronic Modulation of MOF-Derived Co_xMn_yB Nanosheet Arrays toward Efficient Bifunctional Electrocatalysts for Water Splitting. *Journal of Electroanalytical Chemistry* **2024**, *970*, 118553, doi:10.1016/j.jelechem.2024.118553.

70. Han, Z.; Zhang, Y.; Lv, T.; Tan, X.; Wang, Q.; Wang, Y.; Meng, C. Core-Shell Cobalt-Iron Silicide Electrocatalysts with Enhanced Bifunctional Performance in Hydrogen and Oxygen Evolution Reactions. *Journal of Colloid and Interface Science* **2024**, S0021979724027711, doi:10.1016/j.jcis.2024.11.195.

71. Kang, H.; Liu, Y.; Wei, M.; Zhou, L.; Wang, C. Activating Spinel $CoMn_2O_4$ Supported on CNT via Zn Substitution for Bifunctional Oxygen Electrocatalysis. *Journal of Alloys and Compounds* **2024**, *1000*, 175089, doi:10.1016/j.jallcom.2024.175089.

72. Yu, T. FeNi/Ni_2P Nanoparticles Encapsulated in Nitrogen-Doped Porous Carbon: Efficient Electrocatalysts for Oxygen Evolution Reaction. *J Mater Sci.*
